# School self-efficacy is affected by gender and motor skills: findings from an Italian study

**DOI:** 10.7717/peerj.8949

**Published:** 2020-04-29

**Authors:** Roberto Codella, Mariangela Valentina Puci, Matteo Vandoni, Luca Correale, Christel Galvani, Fabio Togni, Francesco Casolo, Alberto Passi, Claudio Orizio, Giampietro Alberti, Fabio Esposito, Cristina Montomoli, Antonio La Torre

**Affiliations:** 1 Department of Biomedical Sciences for Health, Università degli Studi di Milano, Milan, Italy; 2 IRCCS Multimedica, Milano, Italy; 3 Unit of Biostatistics and Clinical Epidemiology, Department of Public Health, Experimental and Forensic Medicine, University of Pavia, Pavia, Italy; 4 Laboratory of Adapted Motor Activity (LAMA), Department of Public Health, Experimental and Forensic Medicine, University of Pavia, Pavia, Italy; 5 Applied Exercise Physiology Laboratory, Department of Psychology, Università Cattolica del Sacro Cuore, Milan, Italy; 6 Department of Educational Studies, Foreign Literacy and Psychology (FORLILPSI), University of Florence, Florence, Italy; 7 Department of Pedagogy, Exercise and Sport Science Degree Course, Università Cattolica del Sacro Cuore, Milan, Italy; 8 Department of Medicine and Surgery, University of Insubria, Varese, Italy; 9 Department of Clinical and Experimental Sciences, University of Brescia, Brescia, Italy

**Keywords:** School self-efficacy, Motor skills, Physical education classes, Children

## Abstract

**Background:**

Perceived school self-efficacy (SE) is an important variable in students’ activities as it affects their motivation and learning. Further, self-efficacy might represent a good predictor of performance, persistence and perseverance. Motor skills and other physical health determinants are extensively debated and linked to cognitive function in children of developmental age. However, inconclusive evidence supports a definitive relationship between perceived school SE and motor skills among schoolchildren. We conducted a cross-sectional study on 6–11-year-old schoolchildren to evaluate the extent by which perceived school SE and physical health determinants were related.

**Methods:**

A SE questionnaire and motor performance battery tests were administered to primary school pupils recruited from 154 sampled schools of northwest Italy. Perceived SE at school was assessed via 12 items from the Caprara’s questionnaire. Motor performance scores were obtained from motor skill tests: 4 × 10 m shuttle run test, SRT; standing broad jump, SBJ; six-minute walking test, 6MWT.

**Results:**

A total of 3,962 children (*M* = 2,019; *F* = 1943) were studied and 68% were normal weight. Overall, a 58% of the sample perceived a high SE, while, as to gender differences, a greater percentage of females perceived high levels of school SE with respect to any other level (χ^2^ = 38.93, *p* < 0.0001). Results from multinomial logistic regression analysis revealed that: (i) females perceived higher SE compared to males; (ii) children who performed better in SRT and 6MWT showed higher levels of perceived school SE; (iii) no significant effect was registered for the body weight. Alternative strategies are encouraged to enhance SE through physical education: structured interventions might enhance both complex motor skills and high-order cognitive skills, like SE, in young children.

## Introduction

Current developments in the fields of behavioral psychology and neurobiology reflect different perspectives about the relationship between motor skills and cognitive skills in prepubertal pupils. Motor and cognitive skills might have comparable developmental timeframes between the ages of 5 and 10 years (Anderson et al., 2001). Sequencing, monitoring, and planning are pivotal for children’s development of either their physical fitness or school motivation (Roebers & Kauer, 2009). Previously, physically active students have been shown to be academically motivated, perceptive and successfully oriented to school tasks (Dwyer et al., 2001). Moreover, other physical health indicators, such as BMI and weight gain, require attention as they may be reciprocally influenced by physical fitness and school self-efficacy (SE) during growth.

In this context, the possible interpolation between pupils’ school SE and their physical fitness needs to be further explored. Self-efficacy plays a decisive role on human behavior in every age group and gender by affecting manifold personal dimensions such as goal setting, aspirations, focus, expectations, and the perception of obstacles or opportunities in the surrounding social (or school) environment. In this regard, perceived SE reflects the level of difficulty students believe they could overcome. Self-efficacy theory postulates that individuals accumulate knowledge to appraise efficacy from four main sources: skilled competences (expertise), observational experiences, social persuasions and emotional arousal (Bandura, 1977). In the Italian questionnaire of perceived school SE (Caprara, 2001), several metacognitive skills are envisaged: planning, that is, when a plan is structured as a hierarchy of sub goals, each requiring actions to master a difficulty and consequently fulfill a task; attention, meant as the ability to discern what it is relevant among numerous cognitive interferences; working memory, which corresponds to the ability of storing and/or handling information over a period of seconds to minutes. In educational settings there has been a lot of research on these constructs, however a cohesive picture linking perceived school SE and motor skills is far to be evidently uncovered among schoolchildren.

The hypothesis was that children with better motor performance would perceive a higher SE. Therefore, the present study aimed at describing perceived school SE and its relationship with motor skills, anthropometrics and age, in 6–11-year-old schoolchildren.

## Materials and Methods

### Subjects and study design

In this cross-sectional study, primary school students (6–11 years old) were recruited from 154 sampled schools in northwest Italy participating at the regional project: “Lombardia in Gioco: a Scuola di Sport”.

In this project, experts in physical education (PE) carried out 20 lessons, 60 min each, once a week, throughout the entire school year, from November 2017 to May 2018. Children with known chronic cardiac, respiratory, neurological or musculoskeletal disorders were excluded. All the described measures were taken at the beginning of the school year, before any PE lesson of the project was given.

### Ethics

This project was funded and approved by the institutional review board of Regione Lombardia (D.g.r. 9 giugno 2017—n. X/6697) along with the Italian National Olympic Committee (CONI). Each of these public bodies approves projects to be conducted on a national level in accordance with nation regulations and guidelines.

The study protocol, including each aspect of the design, was likewise approved by the institutional board of each participating school. All the procedures were compiled in accordance with the Declaration of Helsinki. Written informed consent was obtained from the parents of all participants.

### Measures

Data collection included sociodemographic and anthropometric information (gender, age, weight, height and BMI). Anthropometrics of the children were assessed using standardized techniques, as previously described (Vandoni et al., 2018). Children’s perceived school self-efficacy was evaluated by means of the Caprara’s questionnaire (Caprara, 2001), whereas motor skills were assessed as follows: cardio-respiratory fitness capacity was measured by six-minute walking test (6MWT), musculoskeletal strength was measured by standing broad jump (SBJ), and speed-agility tested by 4 × 10 m shuttle run test (SRT). The motor skill tests used are similar with the tests of ALPHA fitness test battery (Ortega et al., 2015). All tests were applied twice and the best value of two attempts was recorded, except the 6MWT that was conducted only once (Cadenas-Sanchez et al., 2016).

#### Self-efficacy

Pupils’ beliefs on their ability to study certain subjects, their motivation to participate in school-based activities, and the perceived support to their learning, were investigated by means of a validated questionnaire (Caprara, 2001). Twelve items of the questionnaire were scored on a five-point Likert scale (from 1 “totally unable” to 5 “totally-capable”). As previously described (Pastorelli et al., 2001; Falanga, De Caroli & Sagone, 2012), absolute scores of self-efficacy were classified into 3 levels: low self-efficacy (12–40), moderate self-efficacy (41–44) and high self-efficacy (45–60).

#### Aerobic capacity–6MWT

As a valid and reliable functional test for the assessment of aerobic capacity in healthy children (Li et al., 2005; Vandoni et al., 2018), it was performed according to a standardized protocol (ATS Committee on Proficiency Standards for Clinical Pulmonary Function Laboratories, 2002). It consisted in walking for 6 min continuously back and forth through a 20-m long pathway. Participants were encouraged as necessary and stopped if exhausted. The longer the distance covered, the better the performance.

#### Speed and agility—SRT

Participants’ motor fitness was evaluated through the 4 × 10 m shuttle run test, a reliable sport-related functional test evaluating children’s speed, agility, coordination and balance. Procedure was followed as previously described (Artero et al., 2011).

#### Muscular strength—SBJ

Lower body muscular power was assessed through the standing broad jump: a practical, time-efficient and low cost field-test widely adopted in this age group (Artero et al., 2011). It consisted of jumping for the longest distance from a standing position, by using muscular strength and power of the lower limbs.

### Statistical analysis

Data are presented as mean and standard deviations (SD), median with percentiles (p^25^–p^75^) or percentages, as appropriate. Normality of the data was tested with Shapiro–Wilk test.

To evaluate differences between groups, the Student *t* test or Mann–Whitney *U* test were performed for continuous variables, and the χ^2^ test for categorical variables. The Cronbach’s alpha coefficient (Cronbach, 1951) and test–retest via Intraclass Correlation Coefficient (ICC) analyses were done to assess the internal consistency and stability of the scale and motor tests.

In order to explore factors associated with self-efficacy, a multinomial logistic regression was performed using high self-efficacy as the reference category in the model. The results were reported as Relative Risk Ratios (RRRs) and corresponding 95% Confidence Intervals (95% CI). Interactions among sex and age and motor skills were tested, finally the goodness-of-fit of the multinomial model was assessed by Hosmer–Lemeshow test. A *p* value of less than 0.05 was considered significant; all analyses were conducted using STATA/SE for Windows, version 12.

## Results

### Subjects’ characteristics

A total of 3,962 children were recruited in this study, 51% was male and mean age was 8.9 ± 1.4 years. Mean body weight was 31.6 ± 8.8 kg while mean height was 133.2 ± 10.2 cm. Table S1 shows characteristics of overall sample. As shown, 68% of the sample was normal weight, 18% was overweight, 6% was obese and 8% underweight. Table S2 shows distribution of BMI classes (Cole et al., 2000, 2007; WHO, 2018) by gender in different age groups.

### Physical fitness measures

Mean walked distance for all participants was 629.9 ± 91.3 m; SBJ mean value was 124.4 ± 23.6 cm and SRT mean value resulted to be 14.2 ± 2 s. In the comparison between females and males, males performed better as they walked (6MWT) more, jumped (SBJ) more, and ran (SRT) faster than their female peers (Table 1). Test–retest analysis showed a high degree of reliability: ICC was 0.90 for SBJ and 0.93 for SRT (*p* < 0.001).

**Table 1:** Motor skills test results by males and females and in all sample.

	All (*n* = 3,692)	Males (*n* = 2,019)	Females (*n* = 1,943)	*p* Value
6MWT (m)				
Mean ± sd	629.86 ± 91.29	636.85 ± 92.92	622.60 ± 89.02	<0.0001*
Median (p^25^–p^75^)	630 (569–693)	640 (575–700)	620 (562–680)	
SBJ (cm)				
Mean ± sd	124.44 ± 23.66	128.95 ± 23.48	119.75 ± 22.93	<0.0001*
Median (p^25^–p^75^)	124 (109–141)	129 (113–145)	119 (104–135)	
SRT (s)				
Mean ± sd	14.25 ± 1.97	13.99 ± 1.95	14.52 ± 1.96	<0.0001^§^
Median (p^25^–p^75^)	14 (12.91–15.32)	13.7 (12.61–15)	14.24 (13.16–15.6)	

**Notes:**

* *T* di Student test.

§ *U*-Mann Whitney test.

### Perceived school self-efficacy

**Figure 1:**
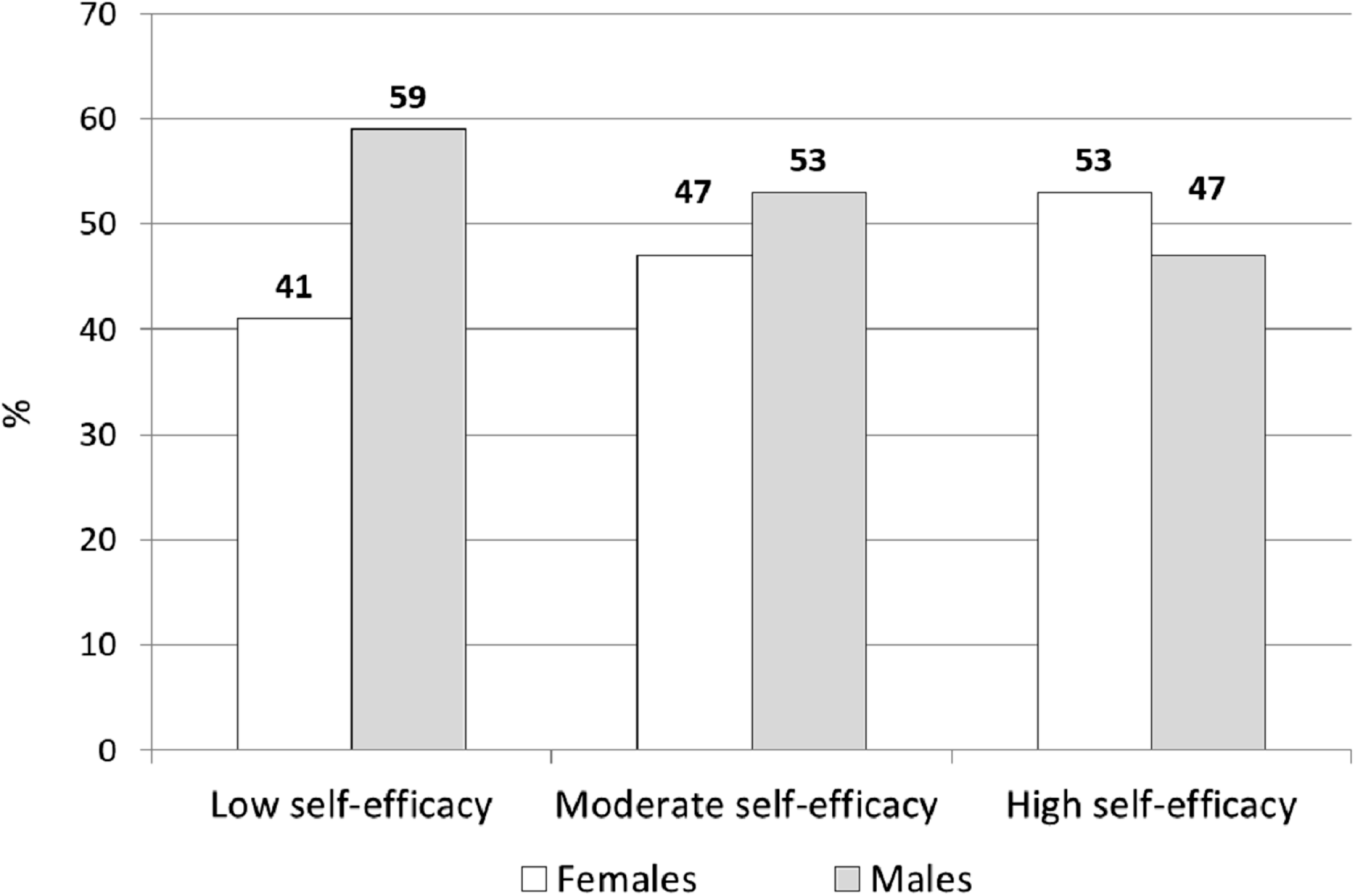
Distribution of males and females by perceived school self-efficacy levels.

Self-efficacy level was high in 58% (*n* = 2,295), low in 25% (*n* = 971) and moderate in 18% (*n* = 696) of all children sampled. Focusing on gender differences, in the high-SE level there was a greater percentage of females than in the other levels (χ^2^ = 38.93, *p* < 0.0001; Fig. 1). The α reliability coefficient was satisfactory for the perceived school SE (α = 0.81) (Altman & Bland, 1996; Tavakol & Dennick, 2011) in the Caprara’s questionnaire, as previously validated. Descriptive statistics of the SE questionnaires were reported, either as overall sample or by gender, in Tables S3, S4 and S5.

### Multinomial logistic regression analysis

According to the results of the multinomial logistic regression analysis (Table 2), the variables associated with both low- and moderate-SE, independently from other characteristics, were: gender (*p* < 0.001), the 6MWT walked distance (low, *p* < 0.001; moderate, *p* = 0.001) and the time used to complete the SRT (low, *p* = 0.003; moderate, *p* = 0.005). Specifically, in males with respect to females, the relative risk of having low-SE vs high-SE was increased by 81% (RRR=1.809 95%CI [1.543–2.122]), while the relative risk of having moderate-SE vs high-SE was increased by 39% (RRR=1.385; 95%CI [1.160–1.650]). For motor skills, as the SRT time increased (by one second), the relative risk of having low-SE vs high-SE was increased by 8% (RRR=1.084; 95%CI [1.027–1.145]), while the relative risk of having moderate-SE vs high-SE was increased by 9% (RRR=1.091; 95%CI [1.026–1.159]). Finally, the increase in 1-m distance walked (6MWT) reduced by 1% the risk of having both low-and moderate-SE vs high-SE (respectively, RRR = 0.996; 95%CI [0.995–0.997] and RRR = 0.9998; 95%CI [0.997–0.999]).

**Table 2:** Variables associated with self-efficacy (SE) in the comparison of children with low-, moderate- and high-SE: multinomial logistic regression analysis (*n* = 3,962).

	RRRs	95% CI	*p*-Value
Low-SE vs high-SE			
Age (year)	1.103	[1.005–1.218]	0.049
Males vs females	1.809	[1.543–2.122]	<0.001
Weight (kg)	1.002	[0.987–1.018]	0.718
Height (cm)	0.998	[0.982–1.016]	0.901
6MWT (m)	0.996	[0.995–0.997]	<0.001
SRT (s)	1.084	[1.027–1.145]	0.003
SBJ (cm)	0.997	[0.992–1.002]	0.303
Moderate-SE vs high-SE			
Age (year)	0.975	[0.872–1.09]	0.650
Males vs females	1.385	[1.160–1.65]	<0.001
Weight (kg)	1.001	[0.984–1.02]	0.917
Height (cm)	1.011	[0.992–1.03]	0.258
6MWT (m)	0.998	[0.997–0.999]	0.001
SRT (s)	1.091	[1.026–1.159]	0.005
SBJ (cm)	0.999	[0.994–1.005]	0.874

Interactions among gender and age and motor skills were tested, but no interaction terms were statistically significant. No other variable was associated with low- or moderate-SE. The Hosmer–Lemeshow goodness-of-fit test indicated that the model fits the data well (χ^2^ = 7.73, *p* = 0.957).

## Discussion

In this study we investigated the relationship between general perceived school self-efficacy and several physical health determinants in 6–11-year-old typically developing children. We found that males perceived more low-to-moderate-SE than females. While slower children in the 4 × 10 SRT perceived more a low-to-moderate-SE, on the contrary children that walked a longer distance in 6MWT perceived a higher SE. In general, females showed a higher sense of perceived SE in school activities with respect to males. On average, males are more likely to support a pro-performance stance (Spaaij et al., 2019). Otherwise, females might settle for lower levels of physical performance (Wood, 1987). However, as motor performance augmented, SE increased across the entire sample. Although past research indicated that females have a higher perception of motivation, ability, performance and self-regulation (Weis, Heikamp & Trommsdorff, 2013; Hyde, 2014), inconclusive results have been reported as to gender differences with regard to school SE.

No significant effect was registered for the body weight, which is reassuring as compared to the extant literature. In fact, male and female children with a lower body mass index tended to show a higher self-efficacy score (Herman et al., 2014; Hjorth et al., 2014) and low physical SE can be worryingly associated to increasing body weight (Steele et al., 2011; Zullig, Matthews-Ewald & Valois, 2016). Other studies have sustained the psychosocial aspect of excessive weight, underlying a negative association between perceived physical SE and BMI in male and female schoolchildren (Carissimi et al., 2017).

Self-efficacy can be pivotal in promoting physical abilities, especially during childhood (Kitzman-Ulrich et al., 2010; Spence et al., 2010). Expertise is the most influential efficacy source (Usher & Pajares, 2006; Usher, 2008; Joët, Usher & Bressoux, 2011; Phan, 2012; Loo & Choy, 2013) as it makes students aware of their capability to accomplish tasks, creating a robust sense of efficacy (Palmer, 2006). In fact, exercise-modulated cognitive abilities and school achievement, as indices of children’s mental function, are often co-stimulated by teachers and educators. However, despite our study highlights an association between motor skills and SE, further research is needed to evaluate the causal mechanisms underlying this important relationship. Alternative strategies to increase self-efficacy through physical education, including parental strategies, might be conceivable. In order to understand the psychosocial aspects influencing self-efficacy, family and social contexts can be studied as critical determinants.

The relevant contribution of motor skills to cognitive learning abilities, such as school achievement, can be explained by the fact that physically active students demonstrate greater attention than sedentary students during classes (Taras, 2005). Besides, pupils who are physically active are likewise those reporting higher level of self-esteem and lower levels of anxiety, which are constitutive elements of an ameliorated self-perception of efficacy and school success.

Self-efficacy is a high-order cognitive skill and may be related to performing complex motor movements. As an important limitation, the motor tasks included in this study may not have aligned with school self-efficacy as it was surveyed. It is possible that the selected motor skills (gross motor skills, non-executive functions) implied a basic cognitive demand. Instead, the motor skills tightly connected with cognitive skills are typically fine, elaborated physical actions requiring demanding cognitive drives. This view has neuropsychological fundaments. In fact, cognitive and motor stimuli are co-initiated by the cerebellum (which is determinant for coordination and elaborated movements) and the prefrontal cortex (which is crucial for high-level cognition). Therefore, the lack of executive function in our design might represent another limitation.

A strength of this study is the very large and cross-sectional sample size of the scholar population, certainly representative of the Italian population given the demographic density and interracial integration of the considered area/community. In fact, students were enrolled from Lombardy—the most densely populated Region of Italy, with highest number of foreign and immigrant people (ISTAT, 2020).

In conclusion, this study explored the relationship between perceived school self-efficacy and underlying sets of motor skills in 6–11-year-old typically developing children. Better motor performances were accompanied by higher perceived school SE, and the higher levels of SE were felt by females.

Future studies should evaluate the relationship between fine motor tasks and higher-order cognitive skills. Particularly, further research might differentiate the contribution of elaborated motor tasks with complex cognitive requirements (e.g., timed movements, bilateral coordination) so to be more successful in higher cognitive engagements, like perceived self-efficacy.

## Conclusions

Across a wide sample of Italian schoolchildren, self-efficacy was greatly perceived by better motor-test performers, especially by females. Whether SE has a clear mediating effect on the achievement of better motor performances remains to be ascertained. The results of this study are still noteworthy within the scenario of PE interventions targeted to enhance both motor performance and intrinsic motivation in children.

## Supplemental Information

10.7717/peerj.8949/supp-1 Supplemental Information 1 School Self Efficacy Questionnaire: Italian and English version.

10.7717/peerj.8949/supp-2 Supplemental Information 2 Raw data.

10.7717/peerj.8949/supp-3 Supplemental Information 3 Characteristics of the study participants.

10.7717/peerj.8949/supp-4 Supplemental Information 4 BMI classes by age and gender of the study participants.

10.7717/peerj.8949/supp-5 Supplemental Information 5 Descriptive statistics of the Italian questionnaire of perceived school self-efficacy in all sample (n=3962).

10.7717/peerj.8949/supp-6 Supplemental Information 6 Descriptive statistics of the Italian questionnaire of perceived school self-efficacy – males (n=2019).

10.7717/peerj.8949/supp-7 Supplemental Information 7 Descriptive results of the Italian questionnaire of perceived school self-efficacy - females (n=1943).

## Abbreviations

6MWT: six-minute walking test
BMI: body mass index
NW: normal weight
OW: overweight
OB: obesity
PE: physical education
SBJ: standing broad jump
SRT: shuttle run test
UW: underweight

## Additional Information and Declarations

### Data Availability

The following information was supplied regarding data availability: The raw data is available in the Supplemental Files.
