# Supplementary material for: School self-efficacy is affected by gender and motor skills: findings from an Italian study
Source: PeerJ. 2020 Apr 29;8:e8949. doi: 10.7717/peerj.8949 (PMC7195827; doi:10.7717/peerj.8949)
Supplement: Supplemental Information 1 [file peerj-08-8949-s001.pdf]

In collaborazione con

PROGETTO "A SCUOLA DI SPORT – LOMBARDIA IN GIOCO III EDIZIONE MOTORIA NELLA SCUOLA PRIMARIA ANNO SCOLASTICO 2017-2018"

QUESTIONARIO A.S.P.

Codice ID

Ti chiediamo ora di indicare, su una scala da 1 (per nulla capace) a 5 (molto capace), il tuo grado di accordo con le seguenti affermazioni.
Non esistono risposte giuste o sbagliate. Rispondi sinceramente, facendo riferimento alla tua personale esperienza.

1	2	3	4	5
**Per nulla capace**	**Poco capace**	**Mediamente capace**	**Abbastanza capace**	**Molto capace**

QUANTO SEI CAPACE DI…

1. Finire in tempo i compiti che ti sono stati assegnati per casa

1	2	3	4	5

2. Impegnarti nello studio quando hai altre cose interessanti da fare

1	2	3	4	5

3. Concentrarti nello studio senza farti distrarre

1	2	3	4	5

4. Prendere appunti delle spiegazioni dell'insegnante

1	2	3	4	5

5. Fare ricerche che ti vengono assegnate utilizzando altri libri (che puoi trovare a casa, in biblioteca ecc.)

1	2	3	4	5

1	2	3	4	5
**Per nulla capace**	**Poco capace**	**Mediamente capace**	**Abbastanza capace**	**Molto capace**

6. Organizzarti nello svolgimento delle attività scolastiche

1	2	3	4	5

7. Programmare le tue attività scolastiche

1	2	3	4	5

8. Ricordare ciò che l'insegnate ha spiegato in classe e ciò che hai letto sui libri

1	2	3	4	5

9. Trovarti un posto dove studiare senza essere distratto

1	2	3	4	5

10. Interessarti alla materie scolastiche

1	2	3	4	5

11. Soddisfare i desideri dei tuoi genitori su ciò che si aspettano da te

1	2	3	4	5

12. Soddisfare le richieste dei tuoi insegnanti

1	2	3	4	5

QUESTIONNAIRE on PERCEIVED SCHOOL SELF-EFFICACY | ID code

Please indicate, on a scale from 1 (“totally unable”) to 5 (“totally-capable”) how much you would agree with the below statements.

There are no “correct” or “wrong” answers. Please respond candidly, based on your own personal experience.

**1**	**2**	**3**	**4**	**5**
**Totally unable**	**Poorly capable**	**Moderately capable**	**Quite capable**	**Totally capable**

HOW MUCH ARE YOU CAPABLE OF…

1. Finish up your homework timely

1 2 3 4 5

2. Get committed in studying when you have other interesting things to do

1 2 3 4 5

3. Get focused on the studying without distractions

1 2 3 4 5

4. Taking notes during teacher’s lecturing

1 2 3 4 5

5. Doing committed research by means of supplementary materials (library-, home-books)

1 2 3 4 5

6. Get organized in running scholastic activities

1 2 3 4 5

7. Planning scholastic activities

1 2 3 4 5

8. Remember what teacher taught or what you read from books

1 2 3 4 5

9. Find a spot where studying without distractions

1 2 3 4 5

10. Get interested in scholastic matters

1 2 3 4 5

11. Meet your parents'expectations on your achievements

1 2 3 4 5

12. Meet your teachers' requests

1 2 3 4 5
