# Supplementary material for: School self-efficacy is affected by gender and motor skills: findings from an Italian study
Source: PeerJ. 2020 Apr 29;8:e8949. doi: 10.7717/peerj.8949 (PMC7195827; doi:10.7717/peerj.8949)
Supplement: Supplemental Information 3 [file peerj-08-8949-s003.docx]

**Table S1.** Characteristics of the study-participants.

|  | All (n=3962) | Males (n=2019) | Females (n=1943) | p-value |
| --- | --- | --- | --- | --- |
| **Age** (years) mean ± SD | 8.9 ± 1.4 | 8.9 ± 1.4 | 8.9 ± 1.5 | 0.4955* |
| **Weight** (kg) mean ± SD | 31.6 ± 8.8 | 32.0 ± 8.8 | 31.1 ± 8.9 | 0.0030* |
| **Height** (cm) mean ±SD | 133.2 ± 10.2 | 133.7 ± 9.9 | 132.8 ± 10.5 | 0.0072* |
| **BMI classes**, n (%) |  |  |  | 0.066§ |
| Underweight (UW) | 334 (8.4) | 147 (7.3) | 187 (9.6) |  |
| Normal weight (NW) | 2682 (67.6) | 1384 (68.6) | 1298 (66.8) |  |
| Overweight (OW) | 722 (18.3) | 370 (18.3) | 352 (18.1) |  |
| Obesity (OB) | 224 (5.7) | 118 (5.8) | 106 (5.5) |  |
|  |  |  |  |  |

Pediatric BMI was stratified according to Cole et al. (Cole et al., 2000, 2007) and divided in classes (WHO, 2018)

*Student t-test, § Pearson Chi-square test
