# Supplementary material for: School self-efficacy is affected by gender and motor skills: findings from an Italian study
Source: PeerJ. 2020 Apr 29;8:e8949. doi: 10.7717/peerj.8949 (PMC7195827; doi:10.7717/peerj.8949)
Supplement: Supplemental Information 4 [file peerj-08-8949-s004.docx]

**Table S2.** BMI classes by age and gender of the study-participants.

| **BMI classes by age** | **Females** | **Males** | **p-value** |
| --- | --- | --- | --- |
| **Age 6** | n (%) | n (%) | 0.367 |
| NW | 180 (73.47) | 179 (78.17) |  |
| OB | 14 (5.71) | 11 (4.80) |  |
| OW | 32 (13.06) | 19 (8.30) |  |
| UW | 19 (7.76) | 20 (8.73) |  |
| **Age 7** |  |  | 0.049 |
| NW | 258 (65.32) | 291 (74.05) |  |
| OB | 23 (5.82) | 13 (3.31) |  |
| OW | 73 (18.48) | 58 (14.76) |  |
| UW | 41 (10.38) | 31 (7.89) |  |
| **Age 8** |  |  | 0.203 |
| NW | 242 (66.12) | 274 (67.16) |  |
| OB | 23 (6.28) | 32 (7.84) |  |
| OW | 62 (16.94) | 75(18.38) |  |
| UW | 39 (10.66) | 27 (6.62) |  |
| **Age 9** |  |  | 0.559 |
| NW | 255 (63.59) | 271 (63.62) |  |
| OB | 27 (6.73) | 29 (6.81) |  |
| OW | 84 (20.95) | 99 (23.24) |  |
| UW | 35 (8.73) | 27 (6.34) |  |
| **Age 10** |  |  | 0.208 |
| NW | 268 (68.02) | 296 (65.63) |  |
| OB | 13 (3.30) | 25 (5.54) |  |
| OW | 74 (18.78) | 96 (21.29) |  |
| UW | 39 (9.90) | 34 (7.54) |  |
| **Age 11** |  |  | 0.659 |
| NW | 95 (66.90) | 73 (65.18) |  |
| OB | 6 (4.23) | 8 (7.14) |  |
| OW | 27 (19.01) | 23 (20.54) |  |
| UW | 14 (9.86) | 8 (7.14) |  |

Chi square test was used for categorial variables.
