# Supplementary material for: School self-efficacy is affected by gender and motor skills: findings from an Italian study
Source: PeerJ. 2020 Apr 29;8:e8949. doi: 10.7717/peerj.8949 (PMC7195827; doi:10.7717/peerj.8949)
Supplement: Supplemental Information 5 [file peerj-08-8949-s005.docx]

**Table S3.** Descriptive statistics of the Italian questionnaire of perceived school self-efficacy in the entire sample (n=3962)

| **How well can you** | **Totally unable** | | **Poorly capable** | | **Moderately capable** | | **Quite capable** | | **Totally capable** | |
| --- | --- | --- | --- | --- | --- | --- | --- | --- | --- | --- |
|  | n | % | n | % | n | % | n | % | n | % |
| 1. Finish up your homework in a timely manner | 34 | 0,86 | 122 | 3,08 | 639 | 16,13 | 1371 | 34,60 | 1796 | 45,33 |
| 2. Get committed to studying when you have other interesting things to do | 140 | 3,53 | 380 | 9,59 | 1144 | 28,87 | 1473 | 37,18 | 825 | 20,82 |
| 3. Get focused on studying without distractions | 159 | 4,01 | 496 | 12,52 | 1125 | 28,39 | 1456 | 36,75 | 726 | 18,32 |
| 4. Taking notes during teacher’s lecture | 1007 | 25,42 | 451 | 11,38 | 883 | 22,29 | 885 | 22,34 | 736 | 18,58 |
| 5. Doing committed research by means of supplementary materials (library-, home-books) | 791 | 19,96 | 373 | 9,41 | 826 | 20,85 | 967 | 24,41 | 1005 | 25,37 |
| 6. Get organized in running scholastic activities | 197 | 4,97 | 208 | 5,25 | 677 | 17,09 | 1389 | 35,06 | 1491 | 37,63 |
| 7. Planning scholastic activities | 299 | 7,55 | 272 | 6,87 | 899 | 22,69 | 1277 | 32,23 | 1215 | 30,67 |
| 8. Remember what teacher taught or what you read from books | 76 | 1,92 | 254 | 6,41 | 817 | 20,62 | 1427 | 36,02 | 1388 | 35,03 |
| 9. Find a spot to study without distractions | 195 | 4,92 | 302 | 7,62 | 706 | 17,82 | 1202 | 30,34 | 1557 | 39,30 |
| 10. Get interested in scholastic matters | 56 | 1,41 | 138 | 3,48 | 635 | 16,03 | 1261 | 31,83 | 1872 | 47,25 |
| 11. Meet your parents’expectations on your achievements | 68 | 1,72 | 144 | 3,63 | 692 | 17,47 | 1426 | 35,99 | 1632 | 41,19 |
| 12. Meet your teachers’ requests | 50 | 1,26 | 155 | 3,91 | 848 | 21,40 | 1483 | 37,43 | 1426 | 35,99 |
