# Supplementary material for: School self-efficacy is affected by gender and motor skills: findings from an Italian study
Source: PeerJ. 2020 Apr 29;8:e8949. doi: 10.7717/peerj.8949 (PMC7195827; doi:10.7717/peerj.8949)
Supplement: Supplemental Information 6 [file peerj-08-8949-s006.docx]

**Table S4.** Descriptive statistics of the Italian questionnaire of perceived school self-efficacy – males (n=2019)

| **How well can you** | **Totally unable** | | **Poorly capable** | | **Moderately capable** | | **Quite capable** | | **Totally capable** | |
| --- | --- | --- | --- | --- | --- | --- | --- | --- | --- | --- |
|  | n | % | n | % | n | % | n | % | n | % |
| 1. Finish up your homework in a timely manner | 18 | 0,89 | 67 | 3,32 | 385 | 19,07 | 668 | 33,09 | 881 | 43,64 |
| 2. Get committed to studying when you have other interesting things to do | 86 | 4,26 | 215 | 10,65 | 624 | 30,91 | 703 | 34,82 | 391 | 19,37 |
| 3. Get focused on studying without distractions | 105 | 5,20 | 284 | 14,07 | 595 | 29,47 | 691 | 34,22 | 344 | 17,04 |
| 4. Taking notes during teacher’s lecture | 535 | 26,50 | 241 | 11,94 | 466 | 23,08 | 429 | 21,25 | 348 | 17,24 |
| 5. Doing committed research by means of supplementary materials (library-, home-books) | 396 | 19,61 | 205 | 10,15 | 460 | 22,78 | 491 | 24,32 | 467 | 23,13 |
| 6. Get organized in running scholastic activities | 99 | 4,90 | 112 | 5,55 | 396 | 19,61 | 704 | 34,87 | 708 | 35,07 |
| 7. Planning scholastic activities | 156 | 7,73 | 158 | 7,83 | 501 | 24,81 | 640 | 31,70 | 564 | 27,93 |
| 8. Remember what teacher taught or what you read from books | 44 | 2,18 | 149 | 7,38 | 423 | 20,95 | 708 | 35,07 | 695 | 34,42 |
| 9. Find a spot to study without distractions | 119 | 5,89 | 184 | 9,11 | 379 | 18,77 | 579 | 28,68 | 758 | 37,54 |
| 10. Get interested in scholastic matters | 35 | 1,73 | 87 | 4,31 | 369 | 18,28 | 629 | 31,15 | 899 | 44,53 |
| 11. Meet your parents’expectations on your achievements | 42 | 2,08 | 95 | 4,71 | 392 | 19,42 | 730 | 36,16 | 760 | 37,64 |
| 12. Meet your teachers’ requests | 28 | 1,39 | 107 | 5,30 | 490 | 24,27 | 748 | 37,05 | 646 | 32,00 |
