# Supplementary material for: School self-efficacy is affected by gender and motor skills: findings from an Italian study
Source: PeerJ. 2020 Apr 29;8:e8949. doi: 10.7717/peerj.8949 (PMC7195827; doi:10.7717/peerj.8949)
Supplement: Supplemental Information 7 [file peerj-08-8949-s007.docx]

**Table S5.** Descriptive results of the Italian questionnaire of perceived school self-efficacy - females (n=1943)

| **How well can you** | **Totally unable** | | **Poorly capable** | | **Moderately capable** | | **Quite capable** | | **Totally capable** | |
| --- | --- | --- | --- | --- | --- | --- | --- | --- | --- | --- |
|  | n | % | n | % | n | % | n | % | n | % |
| 1. Finish up your homework timely | 16 | 0,82 | 55 | 2,83 | 254 | 13,07 | 703 | 36,18 | 915 | 47,09 |
| 2. Get committed in studying when you have other interesting things to do | 54 | 2,78 | 165 | 8,49 | 520 | 26,76 | 770 | 39,63 | 434 | 22,34 |
| 3. Get focused on the studying without distractions | 54 | 2,78 | 212 | 10,91 | 530 | 27,28 | 765 | 39,37 | 382 | 19,66 |
| 4. Taking notes during teacher’s lecturing | 472 | 24,29 | 210 | 10,81 | 417 | 21,46 | 456 | 23,47 | 388 | 19,97 |
| 5. Doing committed research by means of supplementary materials (library-, home-books) | 395 | 20,33 | 168 | 8,65 | 366 | 18,84 | 476 | 24,50 | 538 | 27,69 |
| 6. Get organized in running scholastic activities | 98 | 5,04 | 96 | 4,94 | 281 | 14,46 | 685 | 35,25 | 783 | 40,30 |
| 7. Planning scholastic activities | 143 | 7,36 | 114 | 5,87 | 398 | 20,48 | 637 | 32,78 | 651 | 33,50 |
| 8. Remember what teacher taught or what you read from books | 32 | 1,65 | 105 | 5,40 | 394 | 20,28 | 719 | 37,00 | 693 | 35,67 |
| 9. Find a spot where studying without distractions | 76 | 3,91 | 118 | 6,07 | 327 | 16,83 | 623 | 32,06 | 799 | 41,12 |
| 10. Get interested in scholastic matters | 21 | 1,08 | 51 | 2,62 | 266 | 13,69 | 632 | 32,53 | 973 | 50,08 |
| 11. Meet your parents’expectations on your achievements | 26 | 1,34 | 49 | 2,52 | 300 | 15,44 | 696 | 35,82 | 872 | 44,88 |
| 12. Meet your teachers’ requests | 22 | 1,13 | 48 | 2,47 | 358 | 18,43 | 735 | 37,83 | 780 | 40,14 |
